# Spatial Distribution of Minerals and Selected Bioactive Compounds in White Mold-Ripened and Blue-Veined Cheeses

**DOI:** 10.3390/molecules30183819

**Published:** 2025-09-19

**Authors:** Varineja Drašler, Irena Kralj Cigić, Tomaž Polak, Gregor Marolt, Jernej Imperl, Andreja Čanžek Majhenič, Blaž Cigić

**Affiliations:** 1 Department of Food Science and Technology, Biotechnical Faculty, University of Ljubljana, SI-1000 Ljubljana, Slovenia; varineja.drasler@bf.uni-lj.si (V.D.); tomaz.polak@bf.uni-lj.si (T.P.); 2 Chair of Analytical Chemistry, Faculty of Chemistry and Chemical Technology, University of Ljubljana, SI-1000 Ljubljana, Slovenia; irena.kralj-cigic@fkkt.uni-lj.si (I.K.C.); jernej.imperl@fkkt.uni-lj.si (J.I.); 3 Department of Animal Science, Biotechnical Faculty, University of Ljubljana, SI-1230 Domžale, Slovenia

**Keywords:** mold-ripened cheese, spatial distribution, minerals, spermidine, γ-aminobutyric acid, rind, core

## Abstract

In this study, the contents of minerals, free amino acids (FAAs), biogenic amines (BAs), γ-aminobutyric acid (GABA), and spermidine (SPD) were analyzed in selected white mold-ripened and blue-veined cheeses, including their spatial distribution between rind and core. Blue-veined cheeses contained higher levels of sodium, calcium, phosphorus, FAAs, and SPD. The BAs content was higher in cheeses produced from raw milk. Compared to the cores, the rinds of the analyzed cheeses contained more calcium (up to 66-fold), phosphate (up to 4.4-fold), zinc (up to 9.9-fold), and GABA (up to 17-fold). In white mold-ripened cheeses, where molds do not grow in the core, the rinds also contained more FAAs (up to 15-fold) and SPD (up to 127-fold). Our results confirm previous observations that the rinds of mold-ripened cheeses contain higher amounts of nutritionally valuable cations that form poorly soluble phosphate salts. To our knowledge, this study provides the first demonstration that the rinds of white mold-ripened cheeses are enriched in GABA and SPD, bioactive compounds associated with beneficial health effects. This highlights the high nutritional value of the outer layers of cheese produced with food-grade molds.

## 1. Introduction

Mold-ripened cheeses are highly valued for their unique sensory characteristics. These cheeses undergo a two-stage transformation: an initial lactic acid fermentation of the curd, followed by fungal colonization. In blue-veined cheeses such as Roquefort and Gorgonzola, the milk or curd is inoculated with *Penicillium roqueforti* or *Penicillium glaucum*, and the cheese wheels are pierced to allow inflow of oxygen into the interior, which is essential for mold growth within the cheese matrix [1]. In contrast, *Penicillium camemberti* and *Penicillium candidum* grow only on the surface of white mold-ripened cheeses such as Camembert and Brie, primarily due to low oxygen inflow into the interior, resulting in the formation of a characteristic white cheese surface [2].

The growth of molds leads to profound biochemical and structural changes in the cheese. In both white mold-ripened and blue-veined cheeses, the aerobic metabolism of lactic acid by molds leads to an increase in pH and the development of a pH gradient, with higher pH values in the rind compared to the core [3,4]. This, together with extensive proteolysis [5] and lipolysis [6], has a major impact on texture and contributes to the development of their characteristic sensory properties [7].

Milk and dairy products are important dietary sources of minerals. The main minerals in cheese are calcium, sodium, phosphorus, potassium, and magnesium, while trace elements include zinc, iron, copper, and manganese [8]. During ripening, mineral redistribution occurs within the cheese matrix. In mold-ripened cheeses, elevated surface pH promotes the outward migration and precipitation of calcium and phosphate ions as calcium phosphate (Ca_3_(PO_4_)_2_) crystals on the rind [9]. Other minerals, such as magnesium and zinc, which also form poorly soluble phosphates, may follow similar patterns. However, the spatial distribution of microminerals, particularly zinc, remains insufficiently characterized. Additionally, decreased calcium levels in the core, which destabilize casein micelles, contribute to the characteristic softening of white mold-ripened cheeses during ripening [10].

Starter cultures used in cheesemaking are primarily composed of lactic acid bacteria (LAB), which drive acidification and initiate the structural transformation of curd into cheese. During ripening, increased proteolysis elevates the content of free amino acids (FAAs), which contribute to both the sensory and structural characteristics of cheese [4]. FAAs are also substrates for bacterial decarboxylase enzymes, which lead to the formation of biogenic amines (BAs). BAs are generally considered undesirable in cheese, as their excessive accumulation may cause adverse health effects such as headaches, hypertension, and gastrointestinal disturbances and negatively affect sensory quality through the development of off-flavors [11]. The most common BAs in cheeses are putrescine (PUT), cadaverine (CAD), tyramine (TYM), and histamine (HIM) [11,12]. Their accumulation depends on various factors, including microbial composition, ripening conditions, and hygiene practices [13]. Raw milk cheeses typically contain higher levels of BAs compared to cheeses produced from pasteurized milk [14]. To mitigate health risks, upper limits for the content of BAs in foods, including cheese (less than 200 mg/kg of TYM in cheese; Slovakia), have been established [15]. On the positive side, ripening conditions that promote the accumulation of BAs may also favor the production of beneficial compounds such as γ-aminobutyric acid (GABA) [16]. GABA is essential for regulating the nervous system, as well as for multiple other physiological functions. It is present in certain vegetables and cereals but is most abundant in fermented foods [17]. While LAB used in cheese production generally have low potential to produce harmful secondary metabolites, certain pathogenic strains of *Penicillium roqueforti* [18] and *Penicillium camemberti* [19] have been shown to synthesize mycotoxins under specific conditions. Consumers sometimes remove the rind of white mold-ripened cheeses [20]; however, this is generally unnecessary unless there are signs of secondary mold contamination.

Recently, polyamines (PAs), particularly spermidine (SPD), have gained attention due to their pronounced positive health effects and association with longevity in both animal models [21] and humans [22]. SPD is predominantly found in plant-based foods, while its levels in animal-derived products are generally much lower [23]. This also applies to milk [23,24,25] and most hard and semi-hard cheeses [26], which are relatively poor in SPD. However, mold-ripened cheeses are a much richer source of SPD [27,28,29]. Accordingly, spatial asymmetry in SPD content is expected, especially in white mold-ripened cheeses, due to selective mold growth on the cheese rind, but not in the core. Nevertheless, a recent study found no statistically significant differences between the rind and core of mold-ripened cheeses when no distinction was made between blue and white mold-ripened cheeses [30].

**Figure 1:**
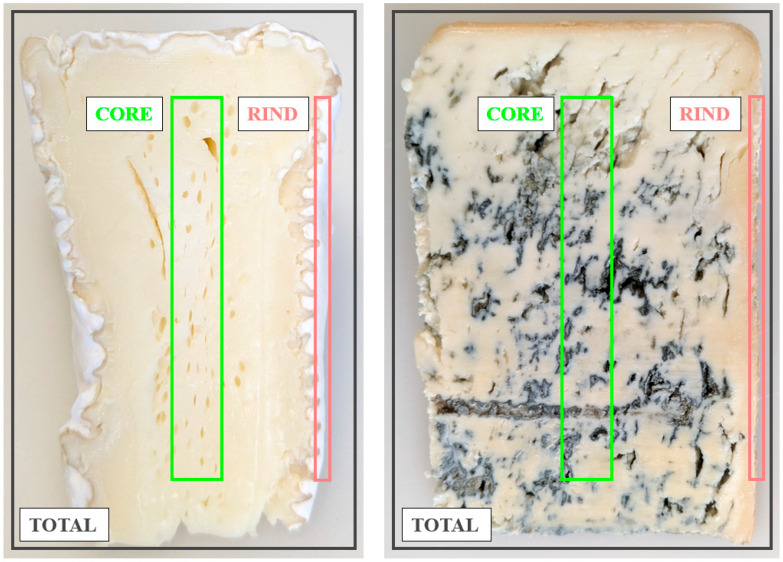
Cheese sampling scheme: total (black), rind (red), and core (green). White mold-ripened cheese is shown on the left, and blue-veined cheese on the right.

In our study, we aimed to investigate the content of minerals, FAAs, BAs, GABA, and SPD in various mold-ripened cheeses, along with their spatial distribution between the rind and core (Figure 1). Additionally, we compared the differences between white mold-ripened and blue-veined cheeses, as well as between cheeses produced from raw and pasteurized milk.

## 2. Results and Discussion

### 2.1. Minerals and Selected Bioactive Compounds in White Mold-Ripened and Blue-Veined Cheeses

Four white mold-ripened (two produced from raw and two from pasteurized milk) and three blue-veined cheeses (one produced from raw and two from pasteurized milk) were thoroughly characterized for their pH, mineral composition (calcium, phosphorus, zinc, sodium, magnesium), free amino acids (20 standard proteinogenic amino acids), GABA, biogenic amines (PUT, HIM, CAD, TYM, tryptamine (TPM), phenylethylamine (PEA)), and polyamines (SPD, spermine (SPM)). All results for the total cheese samples, as well as their rinds and cores, are presented in Tables S1–S4 (Supplementary Materials). Table 1 shows the content of selected minerals and bioactive compounds in the total cheese samples, highlighting notable differences between white mold-ripened and blue-veined cheeses.

A relatively large variation in calcium content was found between the white mold-ripened and blue-veined cheeses, ranging from 2.7 g/kg to 6.9 g/kg (Table 1). Overall, calcium levels were higher in blue-veined cheeses compared to white mold-ripened cheeses. However, previously reported data showed a content of calcium around 4 g/kg in Brie [31] and up to 7.7 g/kg in blue-veined cheeses [1], which is consistent with our findings. The higher calcium content in blue-veined cheeses may be attributed to the addition of calcium chloride, which can be used to improve curd formation yield [1,3], as well as to the specific metabolic activity of molds. In blue-veined cheeses, molds are typically inoculated directly into the milk or during the early stages of curd handling, whereas in the production of white mold-ripened cheeses, the cheese wheels are typically inoculated at later stages, primarily by surface spraying. During ripening, molds metabolize lactic acid, resulting in an increase in pH and promoting calcium retention in the cheese [4].

The content of phosphorus was also higher in blue-veined cheeses (Table 1), with a strong positive correlation (r = 0.96) between calcium and phosphorus levels. This is not surprising, as insoluble colloidal calcium phosphate is one of the predominant forms of calcium in cheeses [32]. The observed phosphorus levels are comparable to those previously reported for Cheddar [33] and blue-veined cheeses [1].

Zinc, along with calcium, is among the most nutritionally important minerals in cheese. A typical 50 g serving can contribute to over 20% of the recommended daily intake of zinc [34]. In this study, zinc contents in mold-ripened cheeses ranged from 22 to 37 mg/kg (Table 1), which is consistent with previously reported values for various types of cheeses [34]. In contrast to calcium and phosphorus, the content of zinc did not differ significantly between white mold-ripened and blue-veined cheeses, and zinc levels did not correlate with phosphate. This is somewhat unexpected, considering that both calcium [35] and zinc [36] form poorly soluble phosphate salts, which typically contribute to mineral retention. One possible explanation is the strong binding affinity of zinc ions to the negatively charged side chains of aspartate and glutamate residues in caseins, which account for a substantial proportion of zinc speciation in cheese [37].

Similar to calcium and phosphorus, sodium content was significantly higher in blue-veined cheeses than in white mold-ripened cheeses (Table 1). Unlike other minerals that primarily originate from milk, sodium in cheese derives mainly from the salting step, as its content in milk is relatively low [38]. The higher sodium content in blue-veined cheeses can be explained by their typical salting processes, dry or wet salting in brine, which enable deep salt penetration and create favorable conditions for the growth of *P. roqueforti* [39].

The content of FAAs was also significantly higher in blue-veined cheeses compared to white mold-ripened cheeses (Table 1). Essential amino acids (AAs) accounted for between 53% and 73% of the total FAAs, with LYS being the predominant AA in most cheeses (compiled from data in Table S3). For Gorgonzola, the FAAs levels observed in this study are consistent with previous findings [40]. The difference in FAAs content between white mold-ripened and blue-veined cheeses can be attributed to the proteolytic potential of molds. *P. roqueforti*, used in blue-veined cheeses, produces potent aspartyl- and metalloproteinases that act not only on the cheese surface, but also within the interior matrix [1]. In contrast, proteolysis in white mold-ripened cheeses is typically limited to surface layers, although prolonged ripening may lead to a further increase in FAAs content. This could be due to the migration of fungal enzymes into the interior and/or the growth of psychrotrophic bacteria during storage [41]. Indeed, two-month refrigerated storage of commercially available Camembert resulted in a fivefold increase in FAAs levels, reaching over 1000 mg/kg [42], placing it within the range of blue-veined cheeses found in this study.

The observed differences in the extent of proteolysis between white mold-ripened and blue-veined cheeses in this study likely influence the pH values, which were on average lower in blue-veined cheeses compared to white mold-ripened cheeses (Table S1). This seems contradictory, as molds metabolize lactic acid into CO_2_ and H_2_O, which are generally associated with increased pH [3,4]. However, the more extensive proteolysis in blue-veined cheeses may counteract this effect, as the hydrolysis of casein can lead to lower pH [43]. Caseins, on average, contain a high proportion of acidic amino acids [44], and the pH of completely hydrolyzed casein is typically below 6 [45].

Despite the higher FAAs levels found in blue-veined cheeses, they did not contain more BAs compared to white mold-ripened cheeses (Table 1). However, significantly higher BAs levels were found in cheeses produced from raw milk. This finding aligns with previous studies showing that raw milk cheeses tend to accumulate more BAs [14]. This is largely related to factors such as microbial composition, ripening conditions, and hygiene practices [13], which appear to influence BAs formation more than the content of amino acid precursors. CAD, PUT, and TYM were quantitatively the most important BAs in cheeses made from raw milk (Table S4). The content of BAs in cheeses produced from pasteurized milk did not exceed 20 mg/kg, reflecting the greater control achievable through thermal treatment of milk. It should be noted that the accumulation of BAs is not strictly linked to a specific cheese type and may vary depending on production and ripening conditions. Nevertheless, raw milk cheeses generally show higher levels of BAs, which may pose a higher potential health risk to consumers; thus, they should be consumed in moderation. This also highlights the practical importance of monitoring BAs levels in cheeses to ensure consumer safety.

A relatively wide span of GABA content was determined in the cheeses analyzed in our study, ranging from 13.1 to 180.1 mg/kg (Table 1). Gorgonzola and Brie made from raw milk showed GABA levels exceeding 80 mg/kg, which is higher than previously found in aged model cheeses produced with inoculated GABA-producing strains [46] and is within a typical range found in various aged cheeses [47]. However, certain blue-veined cheeses or aged hard cheeses can contain much higher contents of GABA, sometimes exceeding 1000 mg/kg [48].

SPD content was significantly higher in blue-veined cheeses compared to white mold-ripened cheeses (Table 1), which is consistent with previous results [49]. Cow’s milk is naturally a poor source of SPD, with reported contents ranging from 0.12 mg/L [50] to 0.3 mg/L [25]. Even assuming complete retention of SPD during cheesemaking, its content in soft cheeses, originating solely from milk (approximately 10 L of milk is needed to produce 1 kg of cheese), would not exceed 3 mg/kg. This is consistent with previous findings showing that half of German cheese samples contain less than 3 mg/kg of SPD [27], SPD was detected in only 3.5% of Swiss hard and semi-hard cheeses [26], and unripened cheeses contain no more than 0.8 mg/kg of SPD [28]. Given the relatively high SPD levels observed in both white mold-ripened and blue-veined cheeses in our study, SPD likely originates from metabolism of the inoculated molds, since most LAB lack the genetic potential to synthesize SPD [51]. However, the literature regarding the origin of SPD in cheese is somewhat inconsistent. Some aged cheeses, even those not deliberately inoculated with molds or yeasts, also contain relatively high levels of SPD [27,30]. For instance, aged Cheddar has been reported to have the highest SPD levels (200 mg/kg) [23]; however, such high contents have not been consistently confirmed by other studies. Secondary contamination of aged cheeses by molds and yeasts may contribute to the accumulation of SPD [26].

### 2.2. Spatial Distribution of Minerals in White Mold-Ripened and Blue-Veined Cheeses

**Figure 2:**
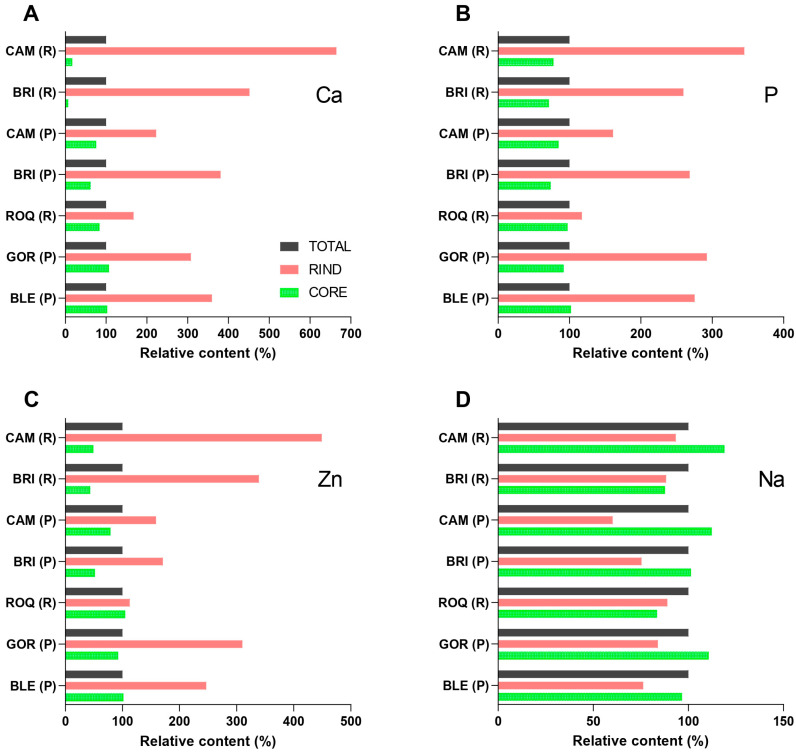
Relative contents of (**A**) calcium, (**B**) phosphate, (**C**) zinc, and (**D**) sodium in the rinds (red) and cores (green) of selected mold-ripened cheeses. Black reference bars indicate the corresponding content in total cheese samples. Cheese abbreviations: CAM, Camembert; BRI, Brie; ROQ, Roquefort; GOR, Gorgonzola; BLE, Bleu de Laqueuille. R = produced from raw milk; P = produced from pasteurized milk.

Figure 2 shows the relative contents of calcium, phosphorus, zinc, and sodium in the rinds and cores compared to the total samples of the analyzed cheeses (Figure 1). For each mineral, the content in the total sample was used as a reference, and thus set to 100% (based on data in Table 1). The values for the rind and core were calculated as percentages of this reference value. Values above 100% indicate a higher content in the rind or core compared to the total cheese sample, whereas values below 100% reflect a lower content. The data used for the calculations for Figure 2 are provided in Table S2.

Substantial variation in the relative calcium distribution was determined for all analyzed cheeses (Figure 2A). The calcium content in the rind of a particular cheese was from 2 to 66 times higher than in the core. The highest calcium rind/core ratio was found in Brie made from raw milk, which also exhibited the largest difference in pH between the rind and core (ΔpH = 1.09) (Table S1). The pH gradient promotes the outward migration of calcium and phosphate ions and their subsequent precipitation in the rind [9]. It has been previously shown that ageing of white mold-ripened cheeses results in softening of the core [52]. The resulting calcium depletion in the core could contribute to softening of the cheese interior [10], as in the case of Brie made from raw milk, where the calcium content in the core was only 0.26 g/kg (Table S2). The calcium rind/core ratio of cheeses was negatively correlated with the total calcium content of cheeses (r = −0.78), indicating that the ratio was higher in cheeses with lower total calcium content. Both a large pH difference between the rind and core and a low total calcium content can therefore contribute to a softer core.

Magnesium, which, similarly to calcium, forms phosphate salts of low solubility, showed a similar distribution pattern. Magnesium content was consistently higher in the rinds than in the cores across all analyzed cheeses (Table S2). While the magnesium rind/core ratios were somewhat lower than for calcium, the correlation between both alkaline earth elements was strong (r = 1.00). The higher levels of magnesium in the rind are consistent with previous findings in Camembert [31,53].

Phosphorus was also significantly more concentrated in the rind of all cheeses (Figure 2B), although the rind/core ratio did not exceed a value of 4.4. The rind/core ratios for phosphorus and calcium in the analyzed cheeses did not show a strong correlation (r = 0.6), which may appear unexpected, given that calcium phosphate precipitation is considered a predominant mechanism underlying the accumulation of both elements in the rind of cheeses. The reason for this discrepancy is likely the lower mobility of phosphate compared to mineral cations, since approximately 40% of total phosphorus in cheese is covalently bound to serine residues in caseins [54,55]. In contrast to colloidal phosphate, protein-bound phosphate exhibits limited solubility and reduced potential for migration, even under extensive proteolysis [54]. Previous studies using a model cheese system have shown that the calcium/phosphorus molar ratio in mineral precipitates ranges from 1.2 to 1.9, depending on the conditions [56]. In our study, the molar calcium/phosphorus ratio (calculated from data in Table S2) was 1.4 ± 0.2 in the rinds and 0.8 ± 0.5 in the cores.

The spatial distribution of zinc in mold-ripened cheeses is not well established, despite this type of cheese being one of the most important dietary sources of zinc in Mediterranean and Western diets. We have found only one SCI manuscript that specifically examined the distribution of zinc between the rind and core of mold-ripened cheeses [53]. Our results are consistent with that study, showing a higher relative content of zinc in the rinds compared to the cores in all analyzed cheese samples (Figure 2C). Although the zinc rind/core ratios were generally lower (not exceeding 9.9) than those observed for calcium, the correlation of rind/core ratios for both minerals was statistically significant (r = 0.88). The zinc content in the rinds of Camembert, Brie, and Gorgonzola exceeded 100 mg/kg, placing them among the richest dietary sources of zinc, since the content of this mineral is higher only in mollusks [57,58]. Additionally, the high protein content of cheese, combined with the absence of antinutritional factors such as phytates, enhances zinc bioaccessibility. Zinc is strongly bound to the side chains of histidine, cysteine, and acidic amino acids [59], which most likely contributes to retention of this mineral within the protein matrix of the core.

In contrast to calcium, phosphorus, and zinc, which tend to accumulate in the rinds of mold-ripened cheeses, sodium shows only minor spatial variation in distribution (Figure 2D). The sodium rind/core ratios ranged from 0.6 to 1.1, indicating that sodium content was, on average, slightly higher in the cores of the cheeses. No significant differences were observed when the white mold-ripened and blue-veined cheeses were analyzed separately, despite the higher total sodium content in the latter. The relatively higher sodium levels in the cores are likely the result of ion counterflow, as sodium diffuses from the rind to the core to balance the outward migration of calcium. During the earlier stages of mold-ripened cheese production, sodium content is initially higher in the rind due to dry or wet salting in brine [1,31].

### 2.3. Spatial Distribution of Free Amino Acids and Their Metabolites in White Mold-Ripened and Blue-Veined Cheeses

**Figure 3:**
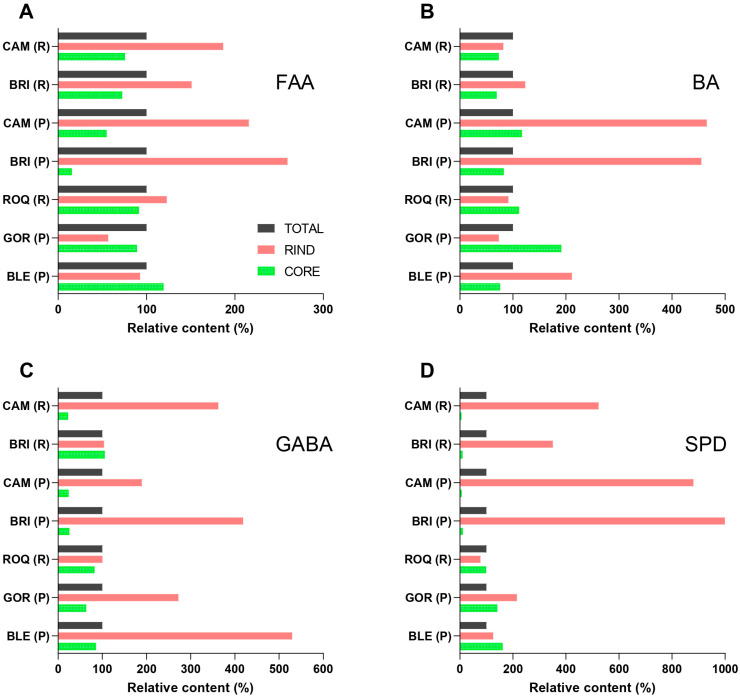
Relative contents of (**A**) free amino acids (FAAs), (**B**) biogenic amines (BAs), (**C**) γ-aminobutyric acid (GABA), and (**D**) spermidine (SPD) in the rinds (red) and cores (green) of selected mold-ripened cheeses. Black reference bars indicate the corresponding content in total cheese samples. Cheese abbreviations: CAM, Camembert; BRI, Brie; ROQ, Roquefort; GOR, Gorgonzola; BLE, Bleu de Laqueuille. R = produced from raw milk; P = produced from pasteurized milk.

Figure 3 presents the relative distribution of FAAs, BAs, GABA, and SPD between the rinds and cores of the cheeses, compared to the total samples. The relative contents were calculated in the same manner as described for mineral contents in Section 2.2. The underlying data used for the calculations are provided in Tables S3 and S4.

The content of FAAs was significantly higher in the rinds compared to the cores (Figure 3A) of white mold-ripened cheeses, while no significant difference in the relative content in the rinds and cores was observed in blue-veined cheeses. This difference in the spatial distribution of FAAs is likely due to the distinct growth patterns and proteolytic capabilities of the molds in each cheese type, as already discussed in Section 2.1. Despite the higher relative FAAs content in the rinds of white mold-ripened cheeses, the absolute FAAs contents in the cores were lower, ranging from 0.5 to 0.8 g/kg, than in the cores of blue-veined cheeses, which contained higher FAAs levels, ranging from 1.4 to 2.3 g/kg (calculated from data in Table S3). This difference can be attributed to the better proteolytic potential of *P. roqueforti* compared to *P. camemberti* [60].

No significant differences were observed in the relative content of BAs in the rinds and cores of the analyzed cheeses (Figure 3B). This indicates that the higher content of amino acid precursors in the rind is not decisive for BAs accumulation. As shown in Table 1, total BAs levels were significantly higher only in cheeses produced from raw milk, where a more diverse and complex microbial community contributes to BAs formation.

The content of GABA was significantly higher in the rind than in the core of all analyzed cheeses (Figure 3C), including when white mold-ripened and blue-veined cheeses were analyzed separately. The highest GABA rind/core ratio (16.8) was found for Brie made from pasteurized milk, while the highest absolute GABA content (340 mg/kg) was observed in the core of Gorgonzola. To the best of our knowledge, there are no available reports related to the spatial distribution of GABA in the rinds and cores of mold-ripened cheeses. GABA is considered one of the most nutritionally beneficial bioactive compounds formed during fermentation [17]. Therefore, its high content may additionally enhance the nutritional value of cheese. GABA is synthesized primarily through the decarboxylation of glutamate by glutamate decarboxylase, while an alternative pathway involves the oxidation of PUT [61]. Higher levels of GABA in the rinds could be associated with more oxidative conditions at the surface, which favor the conversion of PUT to GABA. However, this metabolic pathway is less likely to contribute substantially, due to the limited number of microorganisms with this potential, as well as the lack of a correlation between PUT and GABA contents in the rinds (calculated from data in Tables S3 and S4). Alternatively, the GABA content may also be affected by the catabolic conversion of GABA into the metabolites used in microbial energy metabolism [62].

**Figure 4:**
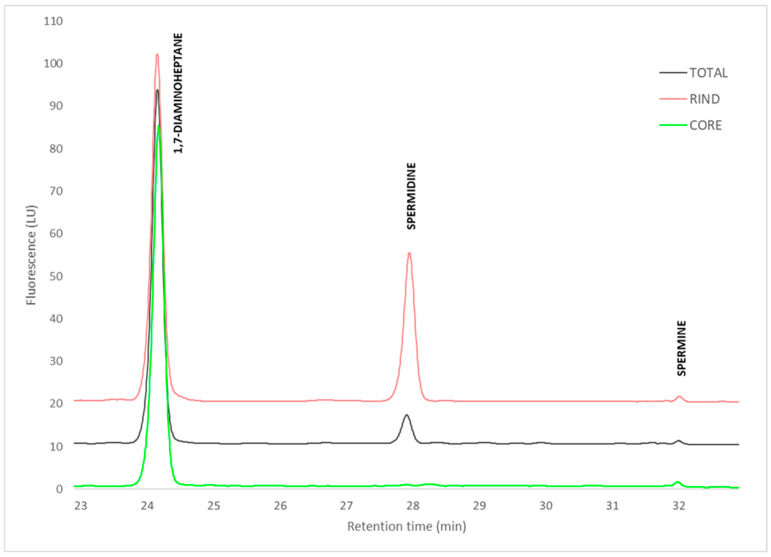
HPLC chromatograms of derivatized (dansyl chloride) samples of Camembert cheese produced from pasteurized milk, extracted with 1,7-diaminoheptane at 10 mg/L in 0.4 M HCl.

SPD exhibited markedly different spatial distribution patterns between white mold-ripened and blue-veined cheeses (Figure 3D and Figure 4). In white mold-ripened cheeses, the SPD rind/core ratios ranged from 38 to 127, indicating high accumulation in the rind. In contrast, the spatial distribution between the rind and core in blue-veined cheeses was not significantly different with regard to total cheese values, with ratios ranging from 0.8 to 1.5. The absolute SPD contents in the rinds of white mold-ripened cheeses (34 ± 15 mg/kg) were comparable to those in the rinds of blue-veined cheeses (38 ± 21 mg/kg) (calculated from data in Table S4). The high SPD rind/core ratios observed in white mold-ripened cheeses are therefore primarily the result of a very low SPD content in their cores (0.4 ± 0.1 mg/kg), whereas SPD contents in the cores of blue-veined cheeses (36 ± 16 mg/kg) were similar to those in the rinds. Our findings support the role of molds as major SPD producers, as SPD distribution clearly follows mold growth patterns in white mold-ripened and blue-veined cheeses. Previous studies examining either combined blue and white mold-ripened cheeses [30] or only blue-veined cheeses [28] found no significant differences in the spatial distribution of SPD between the rind and core. The lack of spatial distribution of SPD in blue-veined cheeses was attributed to its origin from the milk. However, contents in cheese higher than a few mg/kg could hardly originate from milk, due to its low SPD content [24]. Currently, there is no clear evidence regarding which microorganisms are the main producers of SPD in cheese. Nonetheless, it is generally accepted that eukaryotic organisms, such as molds and yeasts, possess a greater potential for SPD biosynthesis compared to bacteria [26]. Therefore, mold-ripened cheeses are often reported to have higher SPD contents than other cheese types [28]. However, the highest SPD levels were determined in certain aged cheeses, such as Cheddar [63] and some Italian cheeses [11], which are not inoculated with molds as part of their production process. The high SPD content in cheeses which were not deliberately inoculated with molds may result from secondary contamination with molds or yeasts. Such uncontrolled microbial growth may pose a food safety risk due to the potential production of mycotoxins [64]. On the other hand, bacterial synthesis of SPD cannot be entirely excluded, especially, again, in the context of secondary contamination, since starter cultures commonly used in cheesemaking are generally not recognized as SPD producers. However, the growth of such non-starter bacteria may also lead to the accumulation of high amounts of undesired BAs [11], which are linked to adverse health effects. In this context, mold-ripened cheeses, especially those produced from pasteurized milk, may offer a favorable nutritional profile. As shown in Table 1, these cheeses typically contain lower levels of BAs and provide higher levels of nutritionally beneficial compounds such as calcium, zinc, GABA, and SPD.

## 3. Materials and Methods

### 3.1. Materials

#### 3.1.1. Cheese

Mold-ripened cheeses were obtained from local supermarkets (Ljubljana, Slovenia), namely four white mold-ripened cheeses––Camembert and Brie (each produced from both raw and pasteurized milk)––and three blue-veined cheeses––Roquefort (produced from raw milk) and Gorgonzola and Bleu de Laqueuille (produced from pasteurized milk). Among them, Camembert (raw milk), Brie (raw milk), Roquefort, and Gorgonzola hold protected designation of origin (PDO) status. For each cheese, two separate packages (from the same production batches) were obtained and independently analyzed. All samples were stored under refrigerated conditions (4 °C) and sampled prior to their expiration dates.

#### 3.1.2. Chemicals

TPM, PHE, PUT, CAD, HIM, TYM, SPD, SPM, and dansyl chloride were obtained from Sigma-Aldrich (St. Louis, MO, USA). l-norleucine, l-glutamine, l-cysteine, skimmed milk powder (ERM-BD150), periodic table mix 1 for ICP, hydrochloric acid (HCl), ammonia solution (NH_3_), sodium carbonate (Na_2_CO_3_), sodium hydroxide (NaOH), pyridine, ethyl acetate, 1-propanol, isooctane, chloroform, methanol, and acetonitrile were obtained from Merck (Darmstadt, Germany). VAR-CAL-1 was obtained from Inorganic Ventures (Christiansburg, VA, USA). The amino acid standard (physiological, A9906) was obtained from Supelco (Bellefonte, PA, USA). Nitric acid (HNO_3_, 67–69% RS-Superpure) was obtained from Carlo Erba Reagents (Milan, Italy). Hydrogen peroxide (H_2_O_2_), ammonium formate, and acetone were obtained from Honeywell (Charlotte, NC, USA). Propyl chloroformate was obtained from Thermo Scientific Chemicals (Waltham, MA, USA). Ultrapure water was obtained using a Milli-Q (MQ) water system (resistivity > 18.2 MΩ cm; Millipore Merck, Darmstadt, Germany).

### 3.2. Methods

#### 3.2.1. Preparation of Cheese Samples

All cheeses were sampled one week before their expiration date. For each cheese product (two separate packages from the same production batches), three types of samples were prepared, representing different structural regions: a combined cross-section (total), the outer part (rind), and the inner part (core), as shown in Figure 1. All samples were collected using a sterile metal scalpel. The total sample consisted of a thin cross-sectional slice ~3 mm thick, encompassing both the rind and core. The rind sample was obtained by slicing a ~2 mm thick layer from the outer surface. The core sample was collected by excising a rectangular cuboid from the interior of the cheese with external faces at least 15 mm underneath the rind. Each sample was then chopped into small pieces approximately 1 mm in size using a scalpel and homogenized with a spatula. Each homogenized material represented a biological replicate. For each cheese type (seven in total), there were two biological replicates for the combined cross-section (total), two for the outer part (rind), and two for the inner part (core). Aliquots of the homogenized samples were weighed and immediately stored at −18 °C for no longer than two weeks until analysis.

The reproducibility of the extraction, derivatization, and chromatography of BAs was determined from the combined cross-section of Brie produced from raw milk. The relative standard deviation (three technical replicates) of any BA did not exceed 7%.

The reproducibility of microwave digestion and ICP-OES elemental analysis was determined using the skimmed milk reference material. The relative standard deviation (five technical replicates) of any of the analyzed elements did not exceed 8%.

#### 3.2.2. Determination of pH Value

To determine the pH value, cheese samples were suspended in MQ water at a 1:10 (*w*/*v*) ratio. The pH of each suspension was measured using a pH meter Testo 206-pH2 (Testo, Titisee-Neustadt, Germany). Before each measurement series, the pH meter was calibrated using a two-point calibration with buffer solutions (pH 4.01 and pH 7.00, at 25 °C) provided by the instrument manufacturer.

#### 3.2.3. Elemental Analysis

Elemental analysis was conducted following acid digestion of the cheese samples using a microwave digestion system (Ethos Up, Milestone, Italy). Approximately 500 mg (exact mass was recorded) of each sample was weighed directly into PTFE vessels, and 8.0 mL of 65% (*w*/*w*) HNO_3_ and 2.0 mL of 30% (*w*/*w*) H_2_O_2_ were added. The vessels were tightly sealed and placed in the microwave digestion system. The digestion temperature program consisted of a gradual increase from room temperature to 200 °C over 30 min, followed by a holding phase at 200 °C for an additional 15 min (from 30 to 45 min), at full power (1800 W). After digestion, the PTFE vessels were left to cool to room temperature. The resulting solutions after digestion were quantitatively transferred into 25.0 mL volumetric flasks and diluted to the mark with MQ water. The blank sample and certified reference material (skimmed milk powder, ERM-BD150) were prepared using the same procedure and analyzed in five independent, parallel samples. Prior to analysis, the samples were further diluted 10-fold and 500-fold with 1% (*v*/*v*) HNO_3_.

The analysis was carried out using an inductively coupled plasma optical emission spectrometer (ICP-OES, Agilent 5100 SVDV, Agilent Technologies, Santa Clara, CA, USA). A forward RF power of 1.2 kW was used, with Ar gas flow rates as follows: nebulizer 0.7 L/min, plasma 12 L/min, and auxiliary 1 L/min. Calibration standards were prepared by diluting two multi-elemental standard solutions, namely periodic table mix 1 for ICP (33 elements, 10 mg/L) and VAR-CAL-1 (4 elements, 100 mg/L), with 1% HNO_3_ solution, covering a concentration range of 0.1 to 1000 µg/L (R^2^ > 0.999). The elements measured were Li, Be, Na, Mg, Al, Si, P, K, Ca, Ti, V, Cr, Mn, Fe, Co, Ni, Cu, Zn, Ga, As, Se, Rb, Sr, Mo, Ag, Cd, In, Sn, Sb, Te, Ba, Tl, Pb, and Bi. Data are presented for the five most relevant minerals (Ca, P, Zn, Na, Mg), for which certified values are listed for the skimmed milk reference material that was analyzed using the same methodology. The determined contents for the reference material ranged from 91% (Ca) to 100% (Na, Zn) of the certified values (Table S2) and did not differ significantly.

#### 3.2.4. Determination of Free Proteinogenic Amino Acids and GABA

FAAs and GABA were analyzed by Phenomenex LC/MS Free (Physiological) Amino Acid Analysis, as described by Uutela et al., with some modifications [65]. Cheese samples were suspended at a 1:10 (*w*/*v*) ratio in 0.4 mol/L HCl containing 10.0 mg/L of the internal standard 1,7-diaminoheptane (IS) and homogenized using a Miccra D-9 Homogenizer Disperser (MICCRA GmbH, Buggingen, Germany) at 21,000 rpm for 1 min at room temperature. The resulting homogenates were centrifuged at 10,000 rcf for 5 min, and the supernatant was collected. The aqueous phase was transferred into microcentrifuge tubes and subjected to derivatization with propyl chloroformate.

A 100 µL aliquot was transferred into glass cryovials, followed by the addition of 50 µL of L-norleucine as an internal standard (50 mg/L in 0.1 M HCl), 120 µL NaOH (0.33 M in 1-propanol), and 80 µL of pyridine (23% in 1-propanol). The mixture was vortexed for 3 min, propyl chloroformate reagent (propyl chloroformate/chloroform/isooctane = 17.4/71.6/11, *v*/*v*/*v*) was added, and the mixture was vortexed for 1 min, allowed to rest for 2 min, and vortexed for 1 min. Derivatives were extracted with 400 µL of ethyl acetate, vortexed, and allowed to separate. If needed, centrifugation (4000 rcf, 5 min) was used to aid phase separation. The upper organic layer (100 µL) was transferred into HPLC vials, and the solvent was evaporated overnight before chromatographic analysis.

FAAs and GABA were determined using high-performance liquid chromatography coupled with tandem mass spectrometry (HPLC-MS/MS) following derivatization. Before injection, derivatized samples were reconstituted with 1000 μL of mobile phase consisting of mobile phase A (1 mM ammonium formate in MQ water) and mobile phase B (1 mM ammonium formate in methanol). Chromatographic separation was performed on an ACQUITY UPLC H-Class PLUS System equipped with a quaternary pump, vacuum degasser, autosampler, and column oven, using a reversed-phase ACQUITY Premier CSH C18 column (1.7 µm, 2.1 × 150 mm; Waters, Milford, MA, USA). The mobile phase gradient began at 40% A and was held for 0.1 min, then linearly decreased to 17% A over 8 min. It was returned to 40% A within 0.1 min and held constant until the end of the run at 12 min. The injection volume was 1.0 μL, the flow rate was set to 0.250 mL/min, and the column temperature was maintained at 35 °C.

Detection was carried out using a Xevo TQ-S micro mass spectrometer (Waters, Milford, MA, USA) equipped with an electrospray ionization (ESI) source operating in positive ion mode (ESI+). The instrument parameters were set as follows: capillary voltage at 1.5 kV, cone voltage at 23 V, source temperature at 150 °C, desolvation gas (nitrogen) temperature at 500 °C with a flow rate of 800 L/h, and cone gas (nitrogen) flow rate of 50 L/h. Detection was performed in Multiple Reaction Monitoring (MRM) mode, targeting specific mass-to-charge (*m*/*z*) ratios and their fragment values for each amino acid and GABA derivatives. Data acquisition and processing were performed using MassLynx software (Target Lynx version 4.2, 2021). The quantification limits (1.0 μL loaded on the column) for proteinogenic amino acids were from 1.6 pg (Leu) to 14 pg (Met, Trp), and the quantification limit for GABA was 2.9 pg.

The data within the Section 2 are presented as total free amino acid (FAAs) content, which refers to the sum of the 20 standard proteinogenic amino acids. GABA was reported separately, as it is non-proteinogenic and is produced through a different metabolic pathway.

#### 3.2.5. Determination of Biogenic Amines and Polyamines

BAs and PAs in cheese samples were determined using a modified method from Kralj Cigić et. al. [66]. The samples used for this analysis were homogenized as described above (Section 3.2.4) and subsequently derivatized with dansyl chloride. Standard BAs solutions were prepared in 0.4 mol/L HCl at a concentration of 5000 mg/L. Mixed calibration standards containing various BAs and the IS were prepared in 0.4 mol/L HCl at concentrations ranging from 0.1 mg/L to 50.0 mg/L.

A 250 µL aliquot of either the sample supernatant or mixed standard solutions was transferred to a 1.5 mL microcentrifuge tube. To this, 50 µL of 2 mol/L NaOH and 75 µL of saturated Na_2_CO_3_ solution were added, and the mixture was vortexed. Next, 500 µL of dansyl chloride solution (10 g/L in acetone) was added, and the reaction mixture was incubated at 40 °C for 45 min on a thermoblock. Following the incubation, 25 µL of 25% NH_3_ solution was added, and the mixture was vortexed again. After 30 min at room temperature, 350 µL of acetone was added, and the solution was mixed thoroughly and centrifuged at 5000 rcf for 5 min. The supernatant was filtered through a 0.45 µm nylon syringe filter into a 1.5 mL glass HPLC vial for subsequent analysis.

An Agilent HPLC system 1100 (Palo Alto, CA, USA) equipped with a degasser, a quaternary pump, an autosampler, and UV-Vis and fluorescent detectors was used. A Kinetex XB-C18 (5 μm, 100 Å, 150 × 4.6 mm) column with a guard column of the same particle size was used (Phenomenex, Torrance, CA, USA). The column temperature was 30 °C; the injection volume was 10 µL; and the mobile phase flow rate was 0.7 mL/min. Eluent A was MQ water, and eluent B was acetonitrile. The initial composition of the mobile phase was 40% B, which changed linearly from 0 to 25 min to 80% B. At 25 to 30 min, a second linear gradient was used to change the mobile phase from 80% B to 100%, where it remained constant until 35 min. Afterwards, the composition changed linearly within 5 min to the initial 40% B. The column was then equilibrated for 2 min. All chromatograms were recorded with sequentially coupled UV-Vis (254 nm) and fluorescence (excitation at 350 nm and emission at 520 nm) detectors. Apart from the elution time, the identity of each chromatographic peak was confirmed by measuring the ratio of the integrals obtained from the fluorescence and UV-Vis detectors. The chromatogram of a real cheese sample obtained with both detectors is shown in Figure S1. Fluorescence signals were used for peak area integration due to better selectivity and sensitivity, except for HIM and TYM, where absorbance at 254 nm was used due to the low fluorescence signal of their dansylated derivatives. All peak areas in the HPLC chromatogram were normalized to those of IS. The median derivatization yield of IS in the complex matrix was 98% (upper quartile 101% and lower quartile 95%). The quantification limits (10.0 μL loaded on the column) for BAs and PAs were from 22 pg (PUT) to 125 pg (TYM).

#### 3.2.6. Statistical Analysis

Statistical analyses were conducted using R-Commander software (version 4.4.1, R Core Team, Vienna, Austria). To compare two independent groups, non-parametric Wilcoxon rank-sum test was used. Spearman’s rank correlation coefficient was used to assess relationships between variables. A *p*-value of ≤ 0.05 was considered statistically significant.

## 4. Conclusions

Mold-ripened cheeses have a characteristic distribution of mineral contents between the core and the rind. Nutritionally important minerals such as calcium, magnesium, and zinc migrate to the outside together with phosphate, so that their content in the rind is several times higher than that in the core. In contrast, the content of sodium, which does not form poorly soluble salts with phosphate, was found to be somewhat higher in the core. The rinds of the mold-ripened cheeses also contain higher levels of the bioactive molecule GABA, which is produced by bacterial enzymes from amino acid precursors. A large difference in the spatial distribution of SPD was found in white mold-ripened cheeses but not in blue-veined cheeses. In Camembert, the SPD content in the rind was more than a hundred times higher than in the core. The high content of SPD in the rind corresponds to the growth pattern of molds that can metabolically synthesize SPD. The rind of mold-ripened cheeses, especially those made from pasteurized milk, has high nutritional value and a low BAs content. A diet rich in SPD and calcium is particularly important for older adults, as endogenous synthesis of SPD decreases with age, and daily calcium requirements increase. This study focused on commercially available products, and our results provide insights into the spatial distribution of bioactive compounds and minerals in mold-ripened cheeses. Future research using model cheese systems under controlled conditions could help to understand other factors that influence their accumulation and distribution.

## Figures and Tables

**Table 1:** Content of calcium (Ca), phosphorus (P), zinc (Zn), sodium (Na), free amino acids (FAAs), biogenic amines (BAs), γ-aminobutyric acid (GABA), and spermidine (SPD) in the total cheese samples.

	White Mold-Ripened					Blue-Veined			
	Raw		Pasteurized		Average	Raw	Pasteurized		Average
	CAM	BRI	CAM	BRI		ROQ	GOR	BLE	
**Ca** (g/kg)	2.7	3.7	5.5	4.5	4 ± 1 *	6.9	6.2	6.1	6.4 ± 0.7 *
**P** (g/kg)	3.1	3.7	4.2	3.5	3.6 ± 0.5 *	4.6	4.4	4.3	4.4 ± 0.2 *
**Zn** (mg/kg)	23.9	31.2	30.5	22.5	27 ± 5	21.5	36.5	29.6	29 ± 7
**Na** (g/kg)	5.9	6.6	6.4	6.1	6.2 ± 0.5 *	11.5	8.5	9.5	9.8 ± 1.4 *
**FAAs** (mg/kg)	404.4	305.3	221.0	189.5	300 ± 100 *	1896.9	2865.4	1518.5	2100 ± 700 *
**BAs** (mg/kg)	1494.6	1884.1	11.0	8.0	900 ± 900	24.6	18.6	10.0	18 ± 7
**GABA** (mg/kg)	25.0	180.8	13.1	51.7	70 ± 70	13.3	126.2	41.1	60 ± 60
**SPD** (mg/kg)	6.5	3.8	5.6	4.8	5 ± 2 *	19.7	26.6	31.9	26 ± 6 *

CAM, Camembert; BRI, Brie; ROQ, Roquefort; GOR, Gorgonzola; BLE, Bleu de Laqueuille. Asterisks (*) indicate statistically significant differences (*p* ≤ 0.05) between white mold-ripened and blue-veined cheeses.

## Data Availability

The original contributions presented in this study are included in the article/Supplementary Material. Further inquiries can be directed to the corresponding author.

## Supplementary Materials

The following supporting information can be downloaded at: https://www.mdpi.com/article/10.3390/molecules30183819/s1. Table S1: pH values of selected mold-ripened cheeses. Table S2: Contents of minerals: calcium (Ca), phosphorus (P), zinc (Zn), sodium (Na), and magnesium (Mg) in selected mold-ripened cheeses. Table S3: Contents of free amino acids (FAAs) and γ-aminobutyric acid (GABA) in selected mold-ripened cheeses. Table S4: Contents of biogenic amines (BAs) and polyamines (PAs) in selected mold-ripened cheeses. Figure S1: The chromatograms of derivatized (dansyl chloride) sample of Camembert cheese produced from pasteurized milk (rind part) obtained by sequentially coupled fluorescence and UV-Vis detectors.

## Abbreviations

The following abbreviations are used in this manuscript:

LAB	Lactic acid bacteria
FAAs	Free amino acids
BAs	Biogenic amines
PUT	Putrescine
CAD	Cadaverine
TYM	Tyramine
HIM	Histamine
GABA	γ-aminobutyric acid
PAs	Polyamines
SPD	Spermidine
TPM	Tryptamine
PEA	Phenylethylamine
SPM	Spermine
CAM	Camembert
BRI	Brie
ROQ	Roquefort
GOR	Gorgonzola
BLE	Bleu de Laqueuille
AAs	Amino acids
R	Produced from raw milk
P	Produced from pasteurized milk
PDO	Protected designation of origin
MQ	Milli-Q
ICP-OES	Inductively coupled plasma optical emission spectrometer
IS	Internal standard 1,7-diaminoheptane
HPLC-MS/MS	High-performance liquid chromatography coupled with tandem mass spectrometry
ESI	Electrospray ionization
