# Supplementary material for: Spatial Distribution of Minerals and Selected Bioactive Compounds in White Mold-Ripened and Blue-Veined Cheeses
Source: Molecules. 2025 Sep 19;30(18):3819. doi: 10.3390/molecules30183819 (PMC12472656; doi:10.3390/molecules30183819)
Supplement: Supplementary file 1 [file molecules-30-03819-s001.zip › molecules-3832736-supplementary.pdf]

Supplementary Materials

Spatial Distribution of Minerals and Selected Bioactive Compounds in White Mold-Ripened and Blue-Veined cheeses

Varineja Drašler [1], Irena Kralj Cigić [2], Tomaž Polak [1], Gregor Marolt [2], Jernej Imperl [2], Andreja Čanžek Majhenič [3], Blaž Cigić [1,*]

[1] Department of Food Science and Technology, Biotechnical Faculty, University of Ljubljana, SI-1000 Ljubljana, Slovenia

[2] Chair of Analytical Chemistry, Faculty of Chemistry and Chemical Technology, University of Ljubljana, SI-1000 Ljubljana, Slovenia

[3] Department of Animal Science, Biotechnical Faculty, University of Ljubljana, SI-1230 Domžale, Slovenia

* Correspondence: blaz.cigic@bf.uni-lj.si (B.C.)

Table S1: pH values of selected mold-ripened cheeses.

Cheese	Sample	**pH**
CAM (R)	Total	6.7 ± 0.1
	Rind	7.3 ± 0.2
	Core	6.5 ± 0.0
BRI (R)	Total	6.4 ± 0.1
	Rind	6.8 ± 0.2
	Core	5.7 ± 0.0
CAM (P)	Total	7.0 ± 0.3
	Rind	6.9 ± 0.0
	Core	6.9 ± 0.1
BRI (P)	Total	6.2 ± 0.0
	Rind	6.6 ± 0.0
	Core	5.7 ± 0.1
ROQ (R)	Total	5.8 ± 0.1
	Rind	5.7 ± 0.1
	Core	5.9 ± 0.1
GOR (P)	Total	6.1 ± 0.0
	Rind	6.2 ± 0.0
	Core	6.1 ± 0.1
BLE (P)	Total	6.5 ± 0.1
	Rind	7.2 ± 0.1
	Core	6.3 ± 0.0

CAM, Camembert; BRI, Brie; ROQ, Roquefort; GOR, Gorgonzola; BLE, Bleu de Laqueuille

R = produced from raw milk; P = produced from pasteurised milk

Table S2: Contents of minerals: calcium (Ca), phosphorus (P), zinc (Zn), sodium (Na), and magnesium (Mg) in selected mold-ripened cheeses.

Cheese	Sample	**Ca (g/kg)**	**P (g/kg)**	**Zn (mg/kg)**	**Na (g/kg)**	**Mg (mg/kg)**
CAM (R)	Total	2.7 ± 0.5	3.1 ± 0.6	23.9 ± 3.3	5.7 ± 0.2	122.4 ± 33.9
	Rind	17.5 ± 0.5	10.5 ± 0.1	107.1 ± 11.4	6.0 ± 0.0	727.5 ± 32.8
	Core	0.4 ± 0.0	2.4 ± 0.1	11.6 ± 0.6	6.9 ± 0.3	48.3 ± 5.1
BRI (R)	Total	3.7 ± 0.3	3.7 ± 0.0	31.2 ± 0.7	6.6 ± 0.3	170.6 ± 0.8
	Rind	16.8 ± 0.5	9.6 ± 1.1	105.9 ± 2.5	7.0 ± 0.2	769.6 ± 129.2
	Core	0.3 ± 0.0	2.6 ± 0.0	13.6 ± 0.2	5.4 ± 0.2	31.4 ± 4.2
CAM (P)	Total	5.5 ± 0.5	4.2 ± 0.1	30.5 ± 1.0	5,7 ± 0.1	238.3 ± 14.7
	Rind	13.0 ± 0.5	6.9 ± 0.2	49.5 ± 1.6	3.9 ± 0.0	356.4 ± 30.6
	Core	4.1 ± 0.0	3.6 ± 0.0	24.1 ± 1.7	6.5 ± 0.2	229.9 ± 0.3
BRI (P)	Total	4.5 ± 1.1	3.5 ± 0.6	22.5 ± 4.6	5.5 ± 0.4	171.9 ± 24.4
	Rind	16.8 ± 0.4	9.3 ± 0.0	37.9 ± 2.8	4.4 ± 0.2	513.3 ± 32.6
	Core	2.8 ± 0.6	2.6 ± 0.3	11.5 ± 1.9	5.3 ± 0.5	125.2 ± 18.2
ROQ (R)	Total	6.9 ± 0.1	4.6 ± 0.1	21.5 ± 0.6	12.4 ± 0.5	296.8 ± 9.8
	Rind	11.5 ± 0.2	5.4 ± 0.2	24.2 ± 0.3	13.6 ± 0.9	321.7 ± 32.3
	Core	5.7 ± 0.3	4.5 ± 0.1	22.4 ± 1.9	11.3 ± 0.3	284.3 ± 2.0
GOR (P)	Total	6.1 ± 1.2	4.4 ± 0.3	36.5 ± 6.5	7.8 ± 1.1	243.9 ± 24.3
	Rind	18.8 ± 2.3	13.0 ± 1.5	113.3 ± 22.3	7.0 ± 1.1	415.1 ± 55.3
	Core	6.4 ± 0.1	4.1 ± 0.2	32.9 ± 0.3	9.2 ± 2.2	232.2 ± 13.4
BLE (P)	Total	6.1 ± 0.3	4.3 ± 0.3	29.6 ± 1.0	8.7 ± 0.6	237.7 ± 15.7
	Rind	21.9 ± 3.2	11.8 ± 1.1	73.0 ± 2.5	8.7 ± 1.8	728.3 ± 38.0
	Core	6.2 ± 0.5	4.3 ± 0.2	30.1 ± 1.7	8.9 ± 0.0	199.1 ± 5.1
Skimmed milk powder (ERM-BD150)	Certified	13.9 ± 0.8	11.0 ± 0.6	44.8 ± 2.0	4.18 ± 0.2	1.26 ± 0.1
	Analysed	12.7 ± 0.5	10.6 ± 0.5	44.3 ± 0.0	4.20 ± 0.2	1.18 ± 0.1

CAM, Camembert; BRI, Brie; ROQ, Roquefort; GOR, Gorgonzola; BLE, Bleu de Laqueuille

R = produced from raw milk; P = produced from pasteurised milk

Table S3: Contents of free amino acids (FAAs) and γ-aminobutyric acid (GABA) in selected mold-ripened cheeses. FAAs includes the following 20 proteinogenic amino acids: His, histidine; Ile, isoleucine; Leu, leucine; Lys, lysine; Met, methionine; Phe, phenylalanine; Thr, threonine; Trp, tryptophan; Val, valine; Arg, arginine; Cys, cysteine; Gln, glutamine; Gly, glycine; Pro, proline; Tyr, tyrosine; Ala, alanine; Asp, aspartic acid; Asn, asparagine; Glu, glutamic acid; Ser, serine. FAA (S) = sum of proteinogenic amino acids.

Cheese	Sample	**His (mg/kg)**	**Ile (mg/kg)**	**Leu (mg/kg)**	**Lys (mg/kg)**	**Met (mg/kg)**	**Phe (mg/kg)**	**Thr (mg/kg)**	**Trp (mg/kg)**	**Val (mg/kg)**	**Arg (mg/kg)**	**Cys (mg/kg)**
CAM (R)	Total	11.2 ± 5.9	69.2 ± 26.9	32.5 ± 10.8	76.9 ± 0.7	0.2 ± 0.2	35.3 ± 6.2	0.1 ± 0.0	0.9 ± 0.6	2.1 ± 1.5	8.3 ± 0.9	-
	Rind	9.2 ± 4.5	83.3 ± 22.1	33.0 ± 7.7	210.3 ± 6.3	0.2 ± 0.2	33.9 ± 7.5	0.3 ± 0.0	0.6 ± 0.3	3.0 ± 1.1	29.5 ± 1.4	-
	Core	12.9 ± 5.5	38.4 ± 12.5	22.0 ± 6.1	101.4 ± 4.2	0.1 ± 0.1	27.1 ± 4.4	0.1 ± 0.0	0.4 ± 0.2	0.8 ± 0.5	1.8 ± 0.3	-
BRI (R)	Total	18.2 ± 1.1	54.5 ± 3.5	29.9 ± 0.7	16.9 ± 2.1	-	38.5 ± 1.3	0.1 ± 0.0	0.9 ± 0.0	2.1 ± 0.1	8.5 ± 2.2	-
	Rind	11.9 ± 3.2	81.8 ± 0.9	34.3 ± 4.2	44.0 ± 11.6	-	45.1 ± 0.2	0.2 ± 0.1	0.3 ± 0.1	3.8 ± 0.3	17.0 ± 3.0	-
	Core	12.7 ± 0.1	30.1 ± 2.7	22.1 ± 0.6	24.0 ± 0.2	-	34.2 ± 2.5	-	0.9 ± 0.1	0.8 ± 0.1	3.6 ± 0.2	-
CAM (P)	Total	0.9 ± 0.6	2.0 ± 0.3	3.0 ± 0.3	126.9 ± 33.8	-	9.8 ± 0.7	0.3 ± 0.1	-	-	11.8 ± 1.1	-
	Rind	2.8 ± 1.0	9.7 ± 0.6	6.3 ± 0.3	267.1 ± 31.3	0.1 ± 0.1	6.1 ± 0.0	0.7 ± 0.1	0.2 ± 0.2	0.4 ± 0.1	14.4 ± 2.1	-
	Core	0.2 ± 0.0	0.9 ± 0.1	2.3 ± 0.1	53.7 ± 9.4	-	13.7 ± 1.0	0.1 ± 0.0	-	-	11.1 ± 0.5	-
BRI (P)	Total	1.5 ± 0.3	12.0 ± 0.2	0.9 ± 0.3	81.3 ± 1.8	-	7.2 ± 1.7	0.2 ± 0.0	-	-	19.7 ± 3.5	-
	Rind	5.8 ± 0.2	9.5 ± 3.6	3.7 ± 0.5	199.0 ± 31.4	-	11.1 ± 2.7	0.5 ± 0.1	-	0.2 ± 0.1	58.7 ± 12.4	-
	Core	-	0.1 ± 0.0	0.3 ± 0.1	16.4 ± 1.7	-	3.5 ± 0.3	-	-	-	1.2 ± 0.2	-
ROQ (R)	Total	25.3 ± 16.4	221.7 ± 27.0	105.3 ± 6.2	774.8 ± 103.7	23.5 ± 6.5	79.9 ± 3.4	2.2 ± 0.3	22.0 ± 4.4	11.0 ± 1.1	70.9 ± 12.3	-
	Rind	21.7 ± 26.6	262.8 ± 4.8	110.0 ± 0.9	960.9 ± 54.6	25.2 ± 0.3	85.0 ± 3.5	2.5 ± 0.1	25.0 ± 3.0	12.9 ± 0.6	79.6 ± 1.1	-
	Core	17.4 ± 0.2	200.8 ± 13.7	95.3 ± 7.3	743.8 ± 150.9	22.8 ± 2.9	68.3 ± 2.7	1.8 ± 0.4	20.9 ± 3.3	9.2 ± 1.8	72.3 ± 10.1	-
GOR (P)	Total	90.7 ± 49.6	344.3 ± 57.7	149.5 ± 33.6	1034.8 ± 67.9	44.1 ± 28.3	145.4 ± 38.4	3.4 ± 0.1	59.8 ± 43.4	19.5 ± 4.2	130.8 ± 20.7	0.2 ± 0.1
	Rind	26.1 ± 3.5	112.2 ± 143.7	86.9 ± 5.7	695.7 ± 35.5	4.2 ± 1.0	87.8 ± 5.0	2.1 ± 0.1	3.1 ± 1.6	10.4 ± 0.4	92.0 ± 8.6	0.1 ± 0.0
	Core	98.0 ± 7.3	10.1 ± 1.1	138.6 ± 5.7	985.8 ± 31.2	36.7 ± 2.4	146.9 ± 1.9	3.8 ± 0.1	83.0 ± 5.8	17.2 ± 0.7	153.1 ± 2.5	0.2 ± 0.0
BLE (P)	Total	38.2 ± 6.7	166.2 ± 30.7	74.2 ± 6.0	728.0 ± 125.8	9.4 ± 2.5	70.9 ± 5.9	1.2 ± 0.1	7.3 ± 1.6	8.1 ± 1.9	68.0 ± 14.8	0.1 ± 0.0
	Rind	16.9 ± 4.6	136.4 ± 21.4	61.7 ± 10.0	668.9 ± 43.5	2.7 ± 1.4	51.0 ± 8.5	0.8 ± 0.0	1.8 ± 0.7	7.1 ± 1.1	60.2 ± 8.6	-
	Core	54.2 ± 9.2	202.2 ± 22.5	83.8 ± 11.4	819.8 ± 22.4	13.4 ± 1.5	79.5 ± 15.1	1.9 ± 0.2	14.7 ± 4.5	9.5 ± 1.3	117.4 ± 5.6	0.1 ± 0.0

CAM, Camembert; BRI, Brie; ROQ, Roquefort; GOR, Gorgonzola; BLE, Bleu de Laqueuille
R = produced from raw milk; P = produced from pasteurised milk
- values below the limit of quantification

Table S3 (Continued)

Cheese	Sample	**Gln (mg/kg)**	**Gly (mg/kg)**	**Pro (mg/kg)**	**Tyr (mg/kg)**	**Ala (mg/kg)**	**Asp (mg/kg)**	**Asn (mg/kg)**	**Glu (mg/kg)**	**Ser (mg/kg)**	**FAA (S) (mg/kg)**	**GABA (mg/kg)**
CAM (R)	Total	25.6 ± 2.5	6.1 ± 3.4	25.0 ± 12.4	67.2 ± 18.5	4.3 ± 4.3	1.0 ± 0.1	1.3 ± 0.0	36.3 ± 8.7	0.8 ± 0.1	404.4 ± 103.9	24.9 ± 7.7
	Rind	98.2 ± 12.0	7.8 ± 2.2	49.9 ± 11.9	95.4 ± 23.2	15.3 ± 7.5	3.0 ± 0.5	5.0 ± 0.9	59.0 ± 0.9	2.4 ± 0.2	739.3 ± 70.7	89.5 ± 21.6
	Core	7.7 ± 0.2	3.4 ± 2.0	10.7 ± 4.7	40.2 ± 8.1	0.8 ± 0.7	0.7 ± 0.2	4.0 ± 0.1	28.8 ± 0.1	0.8 ± 0.1	301.9 ± 49.1	5.3 ± 0.7
BRI (R)	Total	6.0 ± 0.3	5.1 ± 0.5	26.5 ± 1.7	48.9 ± 0.3	4.2 ± 0.2	0.7 ± 0.0	0.5 ± 0.1	43.3 ± 5.4	0.5 ± 0.0	305.3 ± 0.1	180.8 ± 5.8
	Rind	19.5 ± 1.0	8.7 ± 0.2	54.4 ± 3.1	60.2 ± 5.2	19.5 ± 6.6	2.4 ± 0.4	1.3 ± 0.4	55.6 ± 6.8	1.0 ± 0.0	461.1 ± 19.4	187.2 ± 19.0
	Core	4.9 ± 0.2	3.3 ± 0.4	11.9 ± 1.7	40.9 ± 0.9	0.9 ± 0.1	0.4 ± 0.0	1.1 ± 0.2	27.9 ± 3.7	0.4 ± 0.0	220.0 ± 6.3	191.2 ± 25.1
CAM (P)	Total	24.6 ± 8.4	0.1 ± 0.0	1.9 ± 0.5	14.2 ± 2.5	-	1.4 ± 0.4	3.8 ± 0.0	18.7 ± 2.6	1.5 ± 0.3	221.0 ± 51.5	13.1 ± 3.7
	Rind	67.8 ± 4.9	2.1 ± 0.3	9.2 ± 1.0	16.4 ± 0.6	2.2 ± 0.6	4.0 ± 0.2	4.7 ± 0.7	51.3 ± 0.4	3.7 ± 0.0	469.0 ± 44.3	23.9 ± 1.2
	Core	5.8 ± 0.2	-	0.8 ± 0.0	16.4 ± 0.3	-	0.3 ± 0.0	8.0 ± 0.2	5.0 ± 1.3	0.6 ± 0.0	118.8 ± 10.2	3.0 ± 0.1
BRI (P)	Total	31.7 ± 0.1	-	1.3 ± 0.0	13.3 ± 2.3	-	0.7 ± 0.1	1.3 ± 0.3	17.1 ± 2.5	1.2 ± 0.2	189.5 ± 7.9	51.7 ± 2.5
	Rind	102.7 ± 14.4	1.3 ± 0.3	15.2 ± 0.4	29.6 ± 5.5	1.9 ± 0.9	2.5 ± 0.6	6.6 ± 2.0	41.4 ± 4.1	3.4 ± 0.6	493.0 ± 72.6	216.3 ± 3.9
	Core	1.1 ± 0.0	-	0.1 ± 0.0	4.7 ± 0.4	-	0.1 ± 0.0	0.6 ± 0.1	0.3 ± 0.1	0.4 ± 0.0	28.6 ± 2.5	12.8 ± 1.2
ROQ (R)	Total	90.2 ± 13.6	11.5 ± 1.9	63.8 ± 6.4	213.0 ± 0.1	16.5 ± 3.6	7.3 ± 1.0	70.3 ± 15.9	70.4 ± 4.7	17.3 ± 3.4	1896.9 ± 231.5	13.3 ± 1.7
	Rind	102.1 ± 2.0	13.2 ± 0.4	69.9 ± 6.3	338.8 ± 31.4	20.7 ± 0.5	8.4 ± 1.0	83.0 ± 8.3	76.0 ± 2.6	19.3 ± 0.4	2317.0 ± 60.2	13.4 ± 2.3
	Core	78.3 ± 7.6	9.8 ± 1.5	52.5 ± 7.3	167.9 ± 10.0	14.8 ± 2.9	5.3 ± 1.0	76.8 ± 7.3	57.5 ± 6.6	16.4 ± 2.5	1731.6 ± 220.0	10.9 ± 1.0
GOR (P)	Total	89.0 ± 3.6	37.5 ± 11.1	143.2 ± 26.5	349.9 ± 124.9	47.5 ± 15.5	18.8 ± 5.0	27.4 ± 3.2	99.5 ± 19.5	29.8 ± 2.4	2865.4 ± 507.9	126.2 ± 38.3
	Rind	63.3 ± 3.5	18.7 ± 1.6	86.1 ± 4.9	160.1 ± 25.3	30.0 ± 4.2	9.4 ± 0.1	18.7 ± 0.3	56.0 ± 6.5	15.6 ± 1.0	1578.6 ± 227.5	330.2 ± 16.8
	Core	77.5 ± 1.4	36.2 ± 1.3	142.8 ± 9.1	382.8 ± 18.6	35.7 ± 0.7	18.1 ± 0.1	9.7 ± 1.2	99.9 ± 3.5	32.7 ± 0.1	2508.7 ± 19.8	76.0 ± 1.2
BLE (P)	Total	26.8 ± 10.0	14.4 ± 3.6	33.9 ± 9.6	183.1 ± 0.8	14.0 ± 6.1	7.3 ± 1.6	7.9 ± 3.6	49.6 ± 8.1	10.1 ± 2.5	1518.5 ± 212.3	41.1 ± 0.1
	Rind	27.3 ± 3.7	9.8 ± 1.9	36.5 ± 4.5	245.3 ± 1.3	14.3 ± 5.2	6.5 ± 0.7	3.1 ± 0.1	47.5 ± 0.3	4.4 ± 0.8	1402.2 ± 101.0	217.7 ± 2.7
	Core	36.2 ± 10.4	18.7 ± 2.2	44.1 ± 8.8	189.4 ± 40.4	14.4 ± 1.4	9.8 ± 1.0	16.6 ± 1.4	61.5 ± 7.7	18.1 ± 1.4	1805.0 ± 108.2	35.0 ± 3.9

CAM, Camembert; BRI, Brie; ROQ, Roquefort; GOR, Gorgonzola; BLE, Bleu de Laqueuille

R = produced from raw milk; P = produced from pasteurised milk

- values below the limit of quantification

Table S4: Contents of biogenic amines (BAs) and polyamines (PAs) in selected mold-ripened cheeses. BAs include the following compounds: TPM, tryptamine; PEA, phenylethylamine; PUT, putrescine; CAD, cadaverine; HIM, histamine; TYM, tyramine. PAs include spermidine (SPD) and spermine (SPM). BA (S) = sum of all listed biogenic amines.

Cheese	Sample	**TPM (mg/kg)**	**PEA (mg/kg)**	**PUT (mg/kg)**	**CAD (mg/kg)**	**HIM (mg/kg)**	**TYM (mg/kg)**	**BA (S) (mg/kg)**	**SPD (mg/kg)**	**SPM (mg/kg)**
CAM (R)	Total	1.9 ± 0.8	0.1 ± 0.0	403.9 ± 136.2	1009.8 ± 371.7	-	80.9 ± 13.5	1494.6 ± 521.4	6.5 ± 0.1	1.4 ± 0.9
	Rind	1.5 ± 0.3	-	376.0 ± 94.8	801.5 ± 260.5	-	37.3 ± 15.1	1214.8 ± 370.4	33.9 ± 5.6	6.6 ± 0.8
	Core	1.6 ± 0.3	0.2 ± 0.0	278.1 ± 76.7	693.6 ± 181.4	-	101.2 ± 25.7	1072.9 ± 283.8	0.3 ± 0.2	0.8 ± 0.6
BRI (R)	Total	2.0 ± 0.0	0.2 ± 0.1	377.1 ± 14.0	1185.2 ± 86.8	-	321.8 ± 16.0	1884.1 ± 116.9	3.8 ± 0.1	1.3 ± 0.5
	Rind	2.2 ± 0.0	0.6 ± 0.1	458.5 ± 17.7	1436.3 ± 172.2	-	416.1 ± 58.4	2310.9 ± 131.5	13.1 ± 4.7	3.1 ± 1.6
	Core	1.3 ± 0.0	0.1 ± 0.0	273.8 ± 21.2	788.3 ± 120.8	-	239.2 ± 13.1	1301.3 ± 155.1	0.3 ± 0.2	0.9 ± 0.1
CAM (P)	Total	-	-	9.0 ± 2.6	0.4 ± 0.3	-	1.6 ± 0.2	11.0 ± 3.0	5.6 ± 3.1	0.5 ± 0.0
	Rind	-	-	46.4 ± 0.1	2.2 ± 0.6	-	0.8 ± 0.0	49.3 ± 0.4	41.9 ± 1.9	0.8 ± 0.2
	Core	-	-	2.5 ± 0.2	0.2 ± 0.0	-	9.5 ± 1.1	12.2 ± 1.3	0.3 ± 0.0	0.7 ± 0.0
BRI (P)	Total	-	-	5.5 ± 0.7	0.2 ± 0.0	-	2.3 ± 0.8	8.0 ± 0.0	4.8 ± 0.7	2.1 ± 0.3
	Rind	-	-	32.1 ± 4.4	3.0 ± 0.5	-	1.5 ± 0.9	36.6 ± 4.9	48.1 ± 9.7	17.7 ± 3.4
	Core	-	-	2.3 ± 0.1	-	-	4.3 ± 2.1	6.6 ± 2.0	0.5 ± 0.2	0.8 ± 0.0
ROQ (R)	Total	-	1.3 ± 0.0	7.6 ± 0.5	5.9 ± 0.7	-	11.1 ± 2.3	24.6 ± 1.1	19.7 ± 0.2	0.5 ± 0.1
	Rind	-	2.0 ± 0.7	8.2 ± 1.1	6.3 ± 0.3	-	7.3 ± 0.4	21.7 ± 1.0	15.2 ± 0.3	0.4 ± 0.0
	Core	-	1.0 ± 0.3	6.3 ± 0.5	4.3 ± 0.0	-	17.0 ± 7.6	27.7 ± 7.1	19.2 ± 0.6	0.4 ± 0.0
GOR (P)	Total	-	1.8 ± 0.6	16.8 ± 0.1	1.0 ± 0.0	-	0.9 ± 0.0	18.6 ± 0.1	26.6 ± 3.8	1.6 ± 0.9
	Rind	-	1.4 ± 0.1	9.6 ± 0.1	3.4 ± 2.4	-	0.5 ± 0.7	13.5 ± 1.7	57.3 ± 9.2	12.1 ± 1.8
	Core	-	2.0 ± 0.0	35.6 ± 2.4	0.2 ± 0.1	-	1.1 ± 0.6	36.9 ± 1.9	37.3 ± 3.1	0.4 ± 0.0
BLE (P)	Total	-	0.9 ± 0.3	8.2 ± 1.7	1.4 ± 0.8	-	-	10.0 ± 3.0	31.9 ± 0.1	0.9 ± 0.1
	Rind	-	1.2 ± 1.0	17.5 ± 1.0	3.7 ± 1.7	-	-	21.2 ± 2.7	40.0 ± 0.1	7.8 ± 1.0
	Core	-	0.3 ± 0.0	6.5 ± 1.6	0.2 ± 0.3	-	0.6 ± 0.8	7.3 ± 2.1	51.1 ± 6.2	0.6 ± 0.0

CAM, Camembert; BRI, Brie; ROQ, Roquefort; GOR, Gorgonzola; BLE, Bleu de Laqueuille

R = produced from raw milk; P = produced from pasteurised milk

- values below the limit of quantification

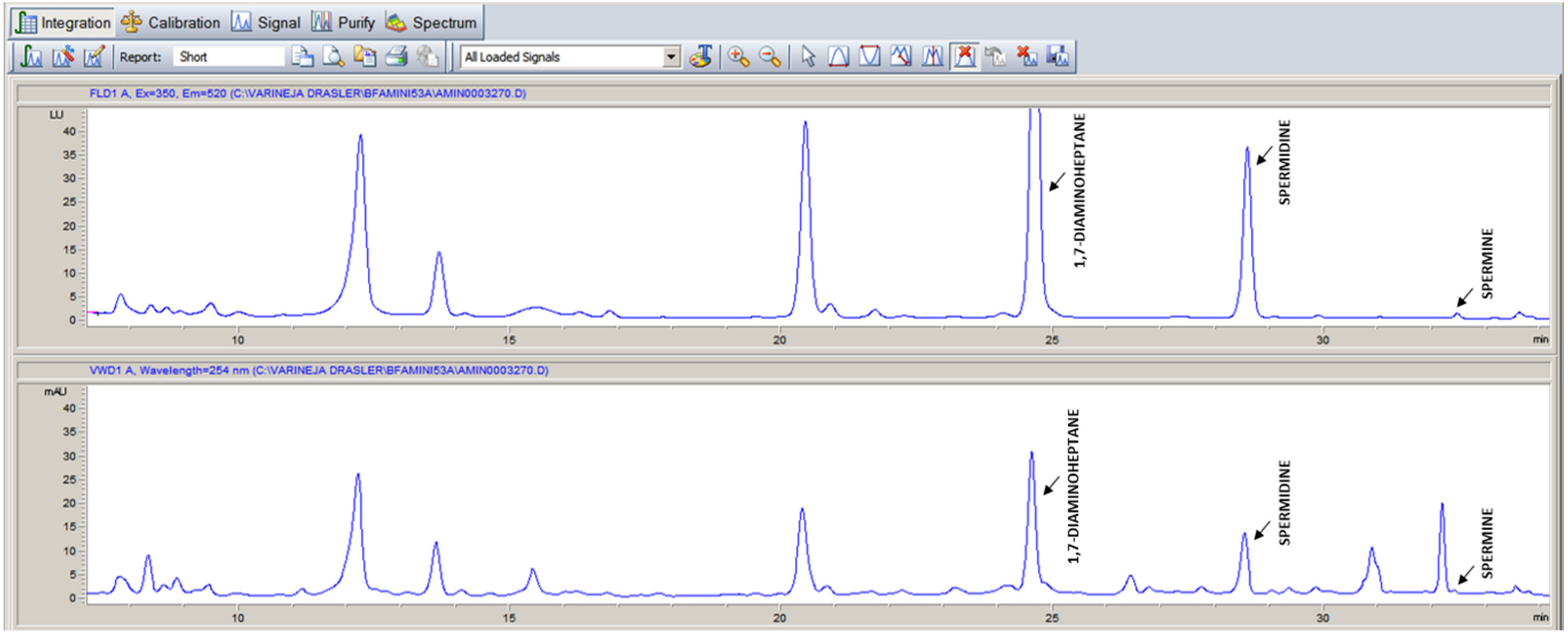

Figure S1: The chromatograms of derivatized (dansyl chloride) sample of Camembert cheese produced from pasteurized milk (rind part) obtained by sequentially coupled fluorescence and UV-Vis detectors
